# Parametric and Nonparametric EEG Analysis for the Evaluation of EEG Activity in Young Children with Controlled Epilepsy

**DOI:** 10.1155/2008/462593

**Published:** 2008-08-04

**Authors:** Vangelis Sakkalis, Tracey Cassar, Michalis Zervakis, Kenneth P. Camilleri, Simon G. Fabri, Cristin Bigan, Eleni Karakonstantaki, Sifis Micheloyannis

**Affiliations:** ^1^Department of Electronic and Computer Engineering, Technical University of Crete, Chania 731 00, Greece; ^2^Institute of Computer Science, Foundation for Research and Technology, Heraklion 71110, Greece; ^3^iBERG: Department of Systems and Control Engineering, Faculty of Engineering, University of Malta, MSD 2080, Msida, Malta; ^4^Faculty of Engineering, Ecological University of Bucharest, 061341 Bucharest, Romania; ^5^Clinical Neurophysiology Laboratory (L. Widen), Faculty of Medicine, University of Crete, Heraklion 71409, Greece

## Abstract

There is an important evidence of differences in the EEG frequency spectrum of control subjects as compared to epileptic subjects. In particular, the study of children presents difficulties due to the early stages of brain development and the various forms of epilepsy indications. In this study, we consider children that developed epileptic crises in the past but without any other clinical, psychological, or visible neurophysiological findings. The aim of the paper is to develop reliable techniques for testing if such controlled epilepsy induces related spectral differences in the EEG. Spectral features extracted by using nonparametric, signal representation techniques (Fourier and wavelet transform) and a parametric, signal modeling technique (ARMA) are compared and their effect on the classification of the two groups is analyzed. The subjects performed two different tasks: a control (rest) task and a relatively difficult math task. The results show that spectral features extracted by modeling the EEG signals recorded from individual channels by an ARMA model give a higher discrimination between the two subject groups for the control task, where classification scores of up to 100% were obtained with a linear discriminant classifier.

## 1. Introduction

Epilepsy is one of the most common human brain disorders. It is often accompanied by disturbances in behavior, brain dysfunction, and cognitive impairment. According to the World Health Organization, 0.7% to 1% of the world's population suffer from epilepsy and this generally peaks in childhood and advanced age, meaning that a large proportion of patients have this chronic disease for most of their lives [1]. This supports the importance of identifying this population as early as possible such that the clinician can prescribe the necessary medication to stop its progression.

Various studies have been carried out to promote our understanding on the development of this disease and on how epileptic subjects differ from normal subjects. Most of the work [2–7] involves the analysis of the electroencephalogram (EEG) which has given a boost to the diagnosis of epilepsy. Complementary studies focusing on the analysis of the heart rate variability have also been presented [8].

Literature indicates that various parametric [9] and nonparametric [10] techniques have been applied to the analysis of epilepsy. Feature extraction and detection methods for EEG signals range from frequency, time-frequency, ARMA based models, to complexity systems modeling, neural networks, and expert systems [11–16]. When analyzing EEG signals in the time domain, abnormal patterns such as spikes and sharp waves are detected; while in the frequency domain, features from the power spectrum are extracted [2, 3, 7]. When comparing seven state-of-the-art approaches for early seizure detection, Jerger et al. showed that there are no significant differences between linear and nonlinear methods [10].

Both in adult epilepsy and child epilepsy, most of the published work is focussed on the seizure itself or on related events such as the ictal, preictal, interictal, postictal parts of the seizure and spikes [17]. Willoughby et al. [2] used the spectral power at 1 Hz interval from 1 Hz to 100 Hz to show whether there are significant differences in the interictal EEG of a group of patients with partial generalized epilepsy. Their results showed that there is a persistent increase in gamma EEG in the absence of epileptic discharges. Jerger et al. [10] have also focussed on interictal spike patterns to predict and ultimately control seizure activity. Using twelve intracranially recorded seizures from four patients, they compared the results of seven linear and nonlinear methods including the analysis of power spectra, cross-correlation, principal components, phase, wavelets, correlation integral, and mutual prediction and showed that all methods were successful in indicating seizure onset before the neurologist, for all but a few seizures. Blanco et al. [11] applied a time-frequency analysis using the Gabor transform to analyze how the traditional frequency rhythms of an EEG signal during an epileptic seizure progress in time. By processing the intracranial recordings obtained for different channels, frequency evolution series were obtained. A systematic calculation of the linear correlation of these series then allowed the possibility of extracting information on how the EEG signals across different regions are related. This together with visual assessment of the EEG and known clinical patient history can aid in identifying the epileptic focus and provide further insight on seizure dynamics.

Patients suffering from epilepsy are most often under the control of antiepileptic drugs (AEDs). The effect of these drugs can also be a reason behind the resulting significant differences between epileptic patients and controls. Salinsky et al. [6] and Tuunainen et al. [7] have both analyzed the effect of adult patients taking AEDs on the spectral analysis of EEG. Salinsky used four occipital EEG measures including the peak frequency, median frequency, relative theta, and delta power to study a group of patients with low-seizure frequency who were either a starting or a stopping AED therapy. A set of cognitive tests and a structured EEG were performed before the AED change and 12–16 weeks after. These results were compared with those of a healthy control group and with patients receiving continuous long-term AED monotherapy. The results showed that the EEG changes of the latter group were not significantly different from the control group. The peak frequency was the most sensitive feature to AED effect for those stopping or starting AEDs as compared to the healthy subjects. For those stopping AEDs, the median frequency and the percentage theta power also gave significant differences. Similarly, Tuunainen et al. used the absolute and relative power as well as the peak power frequency at left occipital brain lobes as features to identify differences between the patient and the control groups. Four-second long, artefact-free EEG epochs of subjects who were instructed to stay awake with their eyes open were analyzed. In this case the results showed that the occipital peak alpha frequency was significantly lower in patients than in controls. Furthermore, the absolute power of the patient group over all electrode sites was significantly higher at baseline than in controls for delta, theta, beta, and total activity. Absolute alpha power was also found to be higher but this result was insignificant.

The discrepancy between the EEGs of epileptic subjects as compared to controls has been studied mostly in adults. Most of the work focuses on the alpha band which is the dominant frequency in the human scalp EEG of adults [5]. Larsson and Kostov [3], for example, considered three 10-second segments, at the beginning, after hyperventilation, and at the end of their artefact-free EEG and analyzed the alpha frequency in 18 epileptic patients as compared to 10 controls. In particular, the peak alpha frequency (PAF) and alpha variations were used as measures to differentiate between the two subject groups. When analyzing children, however, one must keep in mind that the frequency spectrum might not yet be well developed. It is well known that the alpha frequency increases nonlinearly from early childhood to puberty and then starts to decline with age. A seven years old child, for example, will probably show two different peaks in the alpha and theta bands, respectively. This is because at such a young age the alpha peak is still not well defined [5]. Very few literature works have applied the traditional techniques for detecting epilepsy on children. Amongst them is the work of Hongou et al. [4] who investigated the development of the background EEG of 150 epileptic children by using spectral analysis on the recordings from occipital regions. As compared to normal children, their results reflected a significant increase in delta and theta powers together with a decrease of upper alpha power in epileptic children. Different types of epilepsies also resulted in different EEG development [4]. It is therefore quite important to develop methods of high sensitivity and specificity for early detection and categorization of various forms of epilepsy especially in young children.

In this paper, we address the possibility of identifying changes in epileptic subjects versus control subjects at an early stage, when just a few seizures occurred in the past. The epileptic population consists of children selected from the pool of paediatric neurology outpatient clinics of two hospitals in Heraklion, Crete, where they were diagnosed and followed at regular intervals. It should be noted that they were diagnosed with no psychological findings, they were not suffering from severe epileptic syndromes and the visual inspection of their EEG was normal. These children, referred to as controlled epileptic, were put under scrutiny because of their early symptoms, without any detected brain damage; they had one or more epileptic seizures in the past and some of them were under monotherapy with drugs in low doses, without clinical side-effects.

The EEG study of such children compared with matched age controls is important from both the clinical and technical perspectives. Thus, the purpose of this paper is twofold. First, we address the question of whether controlled-epileptic children exhibit spectral differences in their EEGs in comparison to an age-matched control group during the performance of a control task and a mental task. Second, we address the development of a sensitive and reliable measure for discrimination between the two groups. According to our knowledge, such an analysis has not been carried out so far. We compare two different approaches of localizing activity differences and retrieving relevant information for classifying the two populations. In particular, we elaborate the differences in classification results obtained when using a nonparametric signal representation approach such as Fourier transform or wavelets and a parametric signal modeling approach such as autoregressive moving average (ARMA).

The paper proceeds as follows. Section 2 provides all related clinical information for the patients being tested, details on the experimental protocols used for the control and mental tasks considered, and a description of the method used for extracting biomarkers which can be used for classifying children with controlled epilepsy and controls. Section 3 presents the results obtained using a signal representation and a modeling approach and finally, Section 4 presents the discussion of the findings and Section 5 concludes the paper.

## 2. Subjects and Methods

### 2.1. Subjects

The studied population consisted of twenty children aged 9–13 (9 boys, 11 girls) with controlled epileptic seizures, but without any clinical or laboratory findings of brain dysfunction, and twenty (age and sex) matched controls on a volunteer basis. Inclusion criteria for patients and controls consisted of the following: (a) age of 9–13 years old; (b) normal intellectual potential (assessed with WISC-III); (c) absence of neurological damage documented by neurological evaluation for patients and controls and by brain CT and/or MRI scan for patients; and (d) absence of psychiatric problems (based on parent's interview). It should be noted that the EEG signals recorded in both groups were visually evaluated as normal; and detailed clinical, laboratory, and neuropsychological findings could not indicate any population differences; the only clinical indication for the epileptic population was the medical diagnosis of repeated epileptic seizures in the past (the last epileptic event was diagnosed between a few weeks to 1 and half years before this study). These children were treated using common antiepileptic medication only after they exhibit at least two seizures or absences. The types of seizures diagnosed were the most common ones in childhood: Rolandic epilepsy (4 children), idiopathic generalized seizures (5 children), focal seizures (3 children), focal secondary generalized seizures without detectable brain damage (6 children), and absence seizures (2 children). More specifically, the children with absences were free from seizures from the beginning of the treatment with Depakine. The other forms such as generalized tonic-clonic seizures or those with rolandic spikes had a history of two to five episodes, which were prevented after the treatment with common therapeutic (low) dosages of Tegretol. Especially in the case of the absences the treatment is effective from the very beginning and these children were monitored, while treated, for one to two years. During this period no seizures were identified. Absences and idiopathic tonic-clonic seizures are generated from the brainstem, while rolandic seizures are generated from the rolandic area [18]. Apart from eight children (4 generalized and 4 rolandic), the remaining twelve children were treated using Tegretol or Depakine, in small doses without clinical side effects, only after they exhibit at least two seizures or absences. It should be noted that the dosages used for therapeutic purposes are not linked to any known side-effects [19] and the related clinical reports did not diagnose any problems related with visible anatomical damages.

Patients and controls, all right-handed, were individually evaluated in the clinical neurophysiology laboratory, at the Medical School of the University of Crete. All parents of children involved in the study signed a written consent form, after having been informed about the study's purpose and the required procedures. The study was approved by the Local Ethics Committee.

### 2.2. Recordings

**Figure 1:**
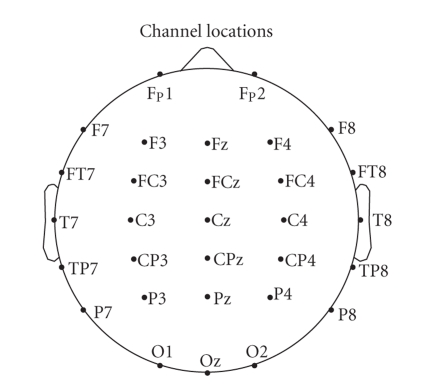
Electrode montage consisting of 30 electrodes placed according to the 10/20 international system.

Continuous EEGs were recorded in an electrically shielded, sound and light attenuated room while participants sat in a reclined chair. The EEG signals were recorded from 30 electrodes placed according to the 10/20 international system, referred to linked A1+A2 electrodes. This electrode montage is shown in Figure 1. The signals were amplified using a set of Contact Precision Instrument amplifiers (Cambridge, Mass, USA, http://www.psylab.com), filtered online with a band pass between 0.1 and 200 Hz, and digitized at 400 Hz and 12 Bits. Offline, the recorded data were carefully reviewed for technical and biogenic artefacts, so that only artefact free epochs of 10.24-second duration were further investigated. Experience obtained from our laboratory and many related to the field publications suggest that this time interval is enough to extract the desired features. Mathematical studies also accept durations ranging from 8 to 12 seconds as indicative of the underlying cognitive task. Artefacts were treated visually by an expert, since many automated artefact removal algorithmic methodologies, even if they are successful in removing certain types of artefacts, they fail to leave physiological EEG intact. Thus, only signal segments without visible artefacts (EOG, EMG, movements) were preserved. For each subject only one representative epoch is included in the data, following the process of intensive visual scrutiny.

### 2.3. Test Description

In this study, two different tasks were analyzed to identify differences in brain dysfunction under tasks with different brain operations. During the control task (Task 1) subjects were at rest and had their eyes fixed on a point displayed on a computer screen to reduce eye artefacts. The second task was a mathematical task (Task 2) involving the subtraction of two digit numbers [20], displayed on an LCD screen located in front of the participants. Such a mental task is considered to be difficult for the studied age group. Stimuli were presented on an LCD screen. Vertical/horizontal eye movements and blinks were monitored through a bipolar montage from the supraorbital ridge and the lateral canthus. The analyzed epochs were acquired during the intensive calculation phase.

### 2.4. Methods

**Figure 2:**
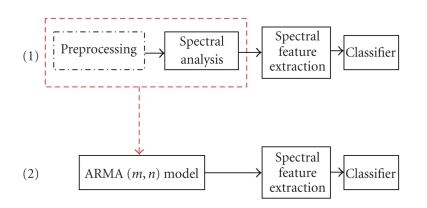
(1) Nonparametric and (2) parametric approaches for feature extraction and classification.

The goal of this analysis is to find discriminating features between epileptic and control children that result in high classification scores. Ideally, a preprocessing step is used to filter out irrelevant data and enhance the discriminating features of the signal. A subsequent spectral analysis step could then be applied to extract those suitable biomarkers. This nonparametric approach, which is labelled as approach (1) in Figure 2, has its limitations. This is mainly due to the fact that the nature and form of the most relevant discriminative features that are to be preserved during the preprocessing step are not known beforehand. In order to avoid degrading the useful part of the signal, our process starts directly from the spectral analysis stage. As an alternative to this scheme, we propose a parametric method that encapsulates both the pre-processing stage and the spectral analysis stage (approach (2)). Using an ARMA model, it is possible to model the dynamics of the EEG signal, without necessarily preserving its detail. Thus, while the first approach performs signal representation, the second approach performs signal modeling.

In order to compare these different approaches in classifying the two subject groups, both methods were implemented and the results obtained were similarly analyzed. For the nonparametric approach both a global Fourier Transform (FT) and wavelets were used for the spectral analysis stage and the biomarkers extracted from each method were compared. The FT gives an average spectral plot over the time period considered. On the other hand, wavelets are mathematical functions that divide the data into different frequency components and then analyze each component with a resolution matched to their scale. Thus, instead of working on a single time or frequency scale, they work on a multiscale basis [21]. Wavelets offer a tradeoff between time and frequency resolution but they are superior to traditional FFT methods when it comes to analyzing data that contains discontinuities and sharp spikes. In addition, the time-windowed version of the wavelets offer a scheme that allows for further refinement of the method in cases where time locked events might be important. When wavelets were compared to the STFT technique [21], the results showed that the STFT is computationally faster but wavelets give more accurate results especially in the detection of epileptic seizures and in EEG signal classification. For these reasons we opted to use wavelets in addition to the application of a global FT to extract power spectral features within predefined frequency bands which are then used for classification purposes.

For the second approach, a time-frequency spectrum is generated using the estimated parameters of the ARMA model derived from each EEG signal. Parametric models are known to enhance the time-frequency resolution of power spectra estimation [9, 22, 23] as they suppress the leakage effect resulting from the used window function [22]. Another advantage of using such a technique is that the ARMA parameters are being estimated at each time instant, thus allowing a more accurate representation of nonstationarities. Once the time-frequency spectra are obtained, spectral features are extracted and fed to the classifier to discriminate between the two subject groups.

#### 2.4.1. Nonparametric Techniques

**Fourier transform (FT)**

The FT transforms a signal in the time domain into its frequency domain representation. By definition, a signal *y*(*t*) has a discrete Fourier transform *Y*(*k*) which is given by [24]

(1) $$Y(k)\;=\;\frac{1}{N}\overset{N-1}{\underset{t=0}{\sum }}y(t)e^{-j2\pi \left(k/N\right)t}.$$

The power spectral density *S* for such a signal is then estimated as shown in (2)

(2) $$S\left(e^{j2\pi \left(k/N\right)}\right)\;=\;\frac{1}{N}{\left|\overset{N-1}{\underset{t=0}{\sum }}y(t)e^{-j2\pi (k/N)t}\right|}^{2}.$$

This spectrum is then used to extract biomarkers which are then fed to a classifier to distinguish between the two populations. Biomarkers are found by calculating the total energy for each of six predefined frequency bands which are the delta (0–4 Hz), theta (4–8 Hz), alpha (8–13 Hz), beta (13–30 Hz), gamma1 (30–45 Hz), and gamma2 (45–90 Hz):

(3) $$M_{B}\;=\;\log_{10}\left(1\;+\;S\cdot f_{S}\right),$$

where *M*_*B*_ is the biomarker at frequency band *B* and *f*_*s*_ is the sampling frequency.

**Wavelet transform (WT)**

Over the past decade, the WT has been developed into an important tool for analysis of time series that contain nonstationary power at many different frequencies (such as the EEG signal), and it has proved to be a powerful feature extraction method [25, 26]. In particular, it has been observed that the epileptic recruitment rhythm during seizure development is well described in terms of the relative wavelet energies [27]. The WT is more suitable for analyzing transient signals because both frequency (scales) and time information can be obtained in good resolution.

The continuous wavelet transform (CWT) was preferred in this work, so that the time and scale parameters can be considered as continuous variables. In the CWT, the notion of scale *s* is introduced as an alternative to frequency, leading to the so-called time-scale representation. The CWT of a discrete sequence *x*_*n*_ with time spacing *δ* *t* and *N* data points (*n* = 0, 1,…, *N* − 1) is defined as the convolution of *x*_*n*_ with consecutive scaled and translated versions of the wavelet function *ψ*_0_(*η*):

(4) $$\begin{array}{ll} W_{n}(s) & \;=\;\overset{N-1}{\underset{n^{\prime }=0}{\sum }}x_{n^{\prime }}{\left(\frac{\delta t}{s}\right)}^{1/2}\psi_{0}^{*}\left[(n^{\prime }\;-\;n)\frac{\delta t}{s}\right], \end{array}$$

(5) $$\begin{array}{ll} \psi_{0}(\eta ) & \;=\;\pi^{-1/4}e^{i\omega_{0}\eta }e^{-\eta^{2}/2}, \end{array}$$

where *η* and *ω*_0_ indicate nondimensional “time” and “frequency” parameters, respectively, $i=\sqrt{-1}$ and (∗) indicates the complex conjugate. In our application, *ψ*_0_(*η*) describes the most commonly used wavelet type for spectral analyses, that is, the normalized complex Morlet wavelet as given in (5). The frequency parameter *ω*_0_ is selected equal to 6 since it is a good tradeoff between time and frequency localization for the Morlet wavelet. The wavelet function *ψ*_0_ is normalized to have unit energy at each scale, so that each scale is directly comparable to each other [25]. The power spectrum of the WT is defined by the square of coefficients in (4) of the wavelet series as |*W*_*n*_(*s*)|^2^. As previously noted, there exists a concrete relationship between each scale and an equivalent set of Fourier frequencies, which for the Morlet wavelet used in this study is given by *f* = 1/(1.03*s*) [28]. The scale set used is given by *s*_*j*_ = *s*_0_2^*jδj*^, *j* = 0,…, *J*, where *s*_0_ = 2*δ* *t* is the smallest scale chosen and *δ* *j* specifies the width of the wavelet function (in our case *δ* *j* = 0.25, meaning that there is a scale resolution of four suboctaves per octave). The largest scale is determined by the value of *J* (in our case *J* = 29, which wraps all six frequency bands of interest).

The first stage of the feature extraction method is based on capturing the time-averaged power spectrum ${\overset{¯}{W}}_{t}^{2}$ for each electrode and scale, which is computed by averaging the power spectrum |*W*_*n*_(*s*)|^2^ over time:

(6) $${\overset{¯}{W}}_{t}^{2}(s)\;=\;(\frac{1}{N})\overset{N-1}{\underset{n=0}{\sum }}{\left|W_{n}(s)\right|}^{2}.$$

Further averaging in scale is performed, in order to map a single feature per frequency band of interest. Thus, the time-scale-averaged power spectrum ${\overset{¯}{W}}_{s,t}^{2}$ is defined as the weighted sum of the time averaged wavelet power spectrum (6) over scales *s*_*j*_1__ to *s*_*j*_2__:

(7) $${\overset{¯}{W}}_{s,t}^{2}\;=\;(\frac{\delta j\delta t}{C_{\delta }})\overset{j_{2}}{\underset{j=j_{1}}{\sum }}\left(\frac{{\left|{\overset{¯}{W}}_{t}(s_{j})\right|}^{2}}{s_{j}}\right),$$

where *C*_*δ*_ is a constant, scale independent factor used for the exact reconstruction of a *δ*(·) function from its wavelet transform (for the Morlet wavelet it equals to 0.776) [28]. Finally, the time-scale-averaged power spectrum ${\overset{¯}{W}}_{s,t}^{2}$ for each of the six frequency bands specified earlier was then calculated as a biomarker, as shown in (8):

(8) $$M_{B}\;=\;\log_{10}\left(1\;+\;{\overset{¯}{W}}_{s,t}^{2}\right),$$

where *B* indicates the selected band.

#### 2.4.2. Parametric Techniques

**Autoregressive moving average (ARMA) modeling**

The autoregressive moving average (ARMA) or Box-Jenkins model is a parametric model where the estimate of the time series at a time instant depends on its past values (deterministic part) and on a random disturbance (stochastic part) [29]. Parametric ARMA models might not result in exact signal reconstruction, but can effectively capture the dynamics of the input process within their time-varying parameters.

**Figure 3:**
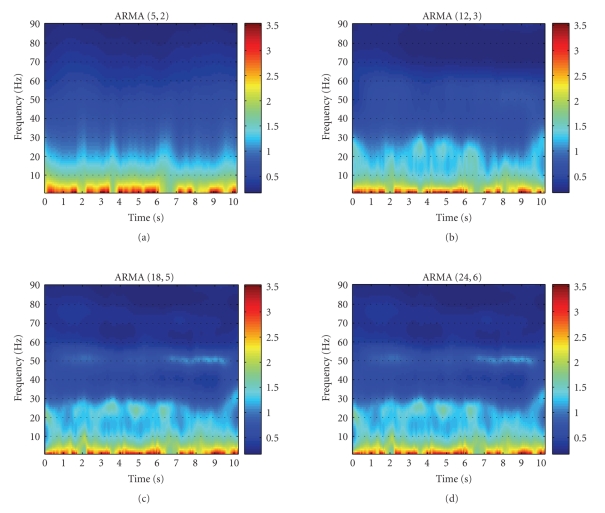
Time-frequency plots of log-power distribution for ARMA model orders (5,2), (12,3), (18,5), and (24,6) are shown.

A parametric method can provide adequate spectral estimates only when the correct model order is chosen. Various techniques have been developed to estimate the optimal order, the most renowned being the Akaike's information criterion (AIC) [30]; however all techniques are based on specific constraints and hence the choice of model order remains questionable. In this analysis, a set of preliminary tests were carried out to find the suitable order which adequately discriminates between the two subject groups. Orders (5,2), (12,3), (18,5), and (24,6) were tested. In Figure 3, plots of the log power spectral values at each frequency and time instant were plotted for the four different model orders. For ARMA (5,2), the resolution of the spectral power is very low (Figure 3(a)) and this results in poor discrimination between the two subject groups. When the model order is increased to (12,3), more features become apparent in the spectral plot (Figure 3(b)) and very low classification of the two groups can be achieved. Higher performance was found with a model order of (18,5). As shown in Figure 3(c), the resolution is substantially improved and superior classification can be achieved, as will be shown later on in this paper. Increasing the model order further gives no significant improvement and this can be seen in Figure 3(c) where the spectral plot at (24,6) is very close to that obtained for an order of (18,5).

An ARMA (*m*, *n*) model [22] is used to model the EEG signals *y*(*t*) recorded at particular electrodes on the scalp, which can be defined as

(9) $$y_{t}\;=\;-\overset{m}{\underset{j=1}{\sum }}a_{t}^{\left(j\right)}y_{t-j}\;+\;\overset{n}{\underset{k=1}{\sum }}b_{t}^{\left(k\right)}e_{t-k}\;+\;e_{t},$$

where *a*_*t*_^(*j*)^ and *b*_*t*_^(*k*)^ are the AR and MA parameters at time instant *t*, respectively, *m* is the number of poles, *n* is the number of zeros, and *e* is a white noise Gaussian process representing the observation error.

Let *θ*_*t*_ be the vector of ARMA parameters and let *ψ*_*t*_ be the regression vector made up of the *m* past signal values and the *n* past observation error values:

(10) $$\begin{array}{c} \theta_{t}\;=\;[-a_{t}^{\left(1\right)},\ldots ,-a_{t}^{\left(m\right)},b_{t}^{\left(1\right)},\ldots ,b_{t}^{\left(n\right)}],\\ \psi_{t}\;=\;[y_{t-1},\ldots ,y_{t-m},e_{t-1},\ldots ,e_{t-n}]. \end{array}$$

The ARMA model in (9) can then be rewritten as

(11) $$y_{t}\;=\;\psi_{t}\theta_{t}^{T}\;+\;e_{t}.$$

If random walk is allowed, the update of the parameter vector can be defined as

(12) $$\theta_{t+1}\;=\;\theta_{t}\;+\;\omega_{t},$$

where *ω*_*t*_ is a normally distributed white noise process with zero mean and covariance matrix *Q*. The set of (11) and (12) represents the structure of a linear state-space formulation, where the model parameters *θ*_*t*_ are also referred to as the states of the system. A Kalman smoother [22] is then used to find an optimal estimate of the time-varying model parameter vector *θ*_*t*_. The advantage of using a smoother rather than a filter is that since data is not being processed in real time, future measurements can be used to find a more accurate estimate of the system parameters at time *t*.

Once an estimate of the ARMA parameters *a*_*t*_^(*j*)^ and *b*_*t*_^(*k*)^ is available, an estimate of the power spectral density can be found using the following equation [22]:

(13) $$P_{t}(f)\;=\;\frac{\sigma_{\varepsilon }^{2}(t)}{f_{s}}\frac{{\left|1+\sum_{k=1}^{n}b_{t}^{\left(k\right)}e^{-i2\pi kf/f_{s}}\right|}^{2}}{{\left|1+\sum_{j=1}^{m}a_{t}^{\left(j\right)}e^{-i2\pi jf/f_{s}}\right|}^{2}},$$

where *σ*_*ε*_^2^(*t*) is the prediction error variance, which in our implementation is assumed to be equal to 1 and *f*_*s*_ is the sampling frequency. In this analysis, the frequency resolution is set to 1 Hz and frequencies from 1 Hz up to 90 Hz were analyzed. As a biomarker, the total energy over the entire time period for each of the six frequency bands, respectively, is then calculated as shown in

(14) $$M_{B}\;=\;\log_{10}\left[1+\overset{f_{2}}{\underset{f_{1}}{\sum }}\overset{T}{\underset{t=1}{\sum }}P_{t}(f)\cdot f_{s}\right],$$

where *M*_*B*_ represents the biomarker for frequency band *B*, *f*_1_, and *f*_2_ represent the range of frequencies falling within band *B*, *t* = 1,…, *T* covers the entire length of data available, *P*_*t*_(*f*) represents the power spectral density for frequency *f* and time instant *t*, and *f*_*s*_ is the sampling frequency. These biomarkers are then used to discriminate between children with controlled epilepsy and control subjects for each of the tasks performed.

### 2.5. Feature Selection

This study proposes a statistical method for mining the most significant lobes, resembling the way many clinical neurophysiological studies evaluate the brain activation patterns. Since the goal is to find significant differences between two groups, the independent two-sample *t*-test is used on the set of biomarkers selected before. The *t*-test assesses whether the means of two populations are statistically different from each other and as a parametric test it assumes that (i) data comes from normally distributed populations, (ii) data is measured at least at the interval level (distance between points on the scale is equal to all parts along the scale), (iii) variances of the populations involved are homogenous; and (iv) all observations are mutually independent [31]. In this analysis, the feature vectors for control subjects (*F*_*C*_) and for epileptic subjects (*F*_*E*_) consist of the biomarkers *M*_*B*_ which are the log-transformed values of the power within a specific frequency band *B* for a particular channel ch. Thus, the feature vectors are formed as

(15) $$\begin{array}{ll} F_{C} & \;=\;\left[M_{B,\text{ch}}^{C1},M_{B,\text{ch}}^{C2},\ldots ,M_{B,\text{ch}}^{C20}\right],\\ F_{E} & \;=\;\left[M_{B,\text{ch}}^{E1},M_{B,\text{ch}}^{E2},\ldots ,M_{B,\text{ch}}^{E20}\right], \end{array}$$

where *M*_*B*,ch_^*C*1^ represents the biomarker for control subject 1 (*C*1), within frequency band *B*, and for a particular channel ch. By using the D’Agostino Pearson test [31] or Kolmogorov-Smrinov's test [32], the features were found to have a normal distribution, thus satisfying assumption (i). Distance between points along the scale of the possible feature values was equal at all parts of the scale, thus ensuring that data is measured at least at the interval level (assumption (ii)). Homogeneity of variances was tested using Levene's test based on the *F*-statistic [32] and in this case it was found that the features from the two populations did not have equal variances. As this violates one of the above assumptions, the *t*-test had to be applied assuming unequal variances (Behrens-Fisher problem). Finally, since the biomarkers in *F*_*C*_ and *F*_*E*_ are coming from two independent populations (controls and epileptics) assumption (iv) is reasonable.

The former statistical analysis technique was used to identify which channels and frequency bands give significant differences between the epileptic subject group and the control group for both the signal representation approaches and the signal modeling approach.

### 2.6. Classification

In this study, the epileptic and control groups were classified by using a strictly linear discriminant analysis (LDA) classifier based on a linear discriminant function that fits a multivariate normal density to each group, with a pooled estimate of covariance as implemented in the MATLAB statistics toolbox [33]. This assumes that the groups can be separated by a linear combination of features, where in the case of two features the boundaries between groups are lines.

## 3. Results

The capabilities of the methods described in Section 2.4 were previously tested on a simulated environment [34], where the spectral content of a simulated signal with known spectral activity was estimated. All methods successfully detected the characteristics of the signal, thus proving that they are appropriate for the analysis of real band-limited signals.

During the tasks performed, neurological examinations showed that there are no differences in achievement between children younger than 11 years old and children in the age of 11 and above. Therefore, in subsequent analyses the subjects were not divided into different age groups. Both nonparametric and parametric approaches were then applied to the real EEG data, where each signal was initially set to zero mean and unit variance. In each case, we compute the channel/band significance, as well as the corresponding classification scores with sensitivity-specificity measures.

**Figure 4:**
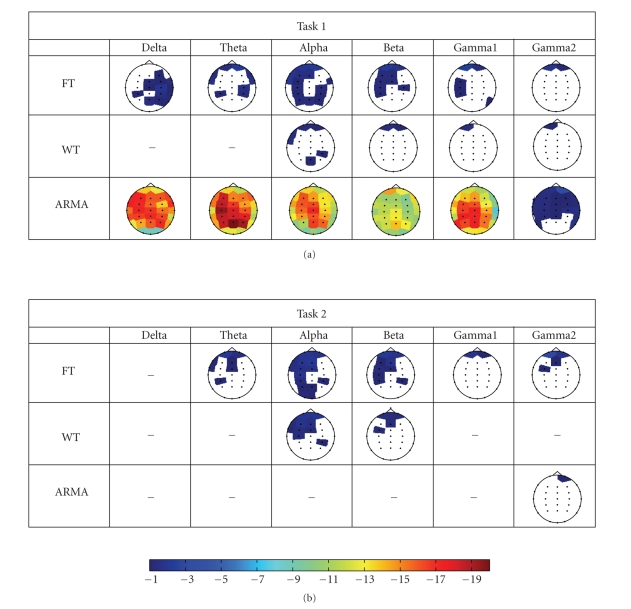
Topographic maps showing the *p*-values for Task 1. The black dots in each image represent the channel locations. Lower *p*-values are indicated in shades of red while *p*-values close to the threshold of 0.1 are indicated in shades of blue. Blank areas within each topographic map indicate that the features extracted from that particular channel do not give significant differences between the two populations (*P* > 0.1).

**Figure 5:**
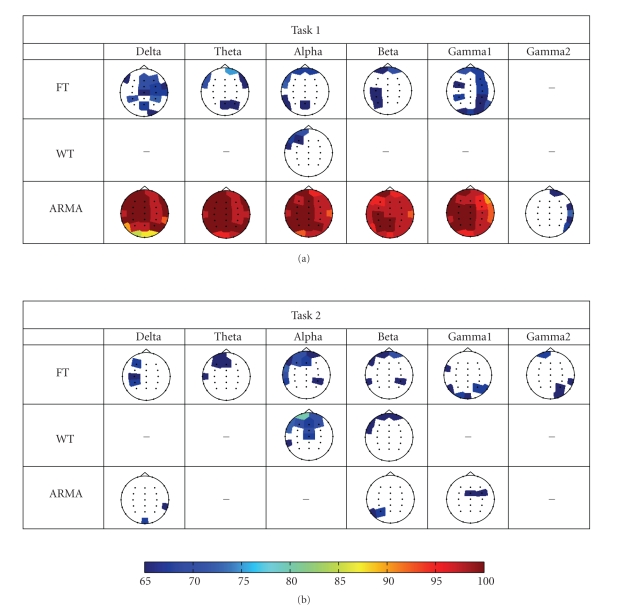
Topographic maps showing the classification scores above 65% over all 27 channels.

Figure 4 illustrates the topographic maps of the log-transformed *p*-values between the two populations, obtained for each method, task, and frequency band. Cells which have been left blank indicate no significant difference at the 90% confidence interval (i.e., *p* > 0.1). All shaded channel locations represent a *p*-value less than 0.1, with shades of red indicating the lowest *p*-values. From these topographic maps, it is clear that for the control task (Task 1), the ARMA model has effectively modeled the EEG signals as to be able to extract spectral features that depict significant differences between the epileptic children and the control group. Even if these differences are attributed to drugs, they are still highly significant, with *p*-values being in the order of 10^−19^. The topographic plots show that the gamma2 band (45–90 Hz) has *p*-values closer to the threshold, and posterior channels are not found to be significant. In all other bands significant differences are distributed over the entire brain region. From these latter frequency bands, the occipital region reflects the least discrimination power (higher *p*-values) between the groups from all significant channels. This is also illustrated in the topographic maps of classification scores shown in Figure 5 where occipital channels gave the lowest scores from all other significant channels. This figure also shows that the nonparametric approaches, as compared to the ARMA approach, gave lower classification scores. The global FT approach resulted in a larger number of channels showing significant differences between the populations, whereas the WT approach seems to be more selective (see Figure 4). Left and right brain areas dominated in most frequency bands but much weaker significance levels were obtained when compared to the ARMA approach. In most cases frontal channels were also found to be significant but one must keep note that frontal channels may be affected by eye movements and can thus result in sporadic discrimination. For this task and for the type of biomarker considered here, the WT features in most bands do not show much significant discrimination. The largest difference was found in frontal channels and in some posterior channels within the alpha band. The global FT was also able to identify the same regions as the WT did, but with higher *p*-values (much weaker significance levels) than the ARMA counterpart. These results indicate the weakness of the nonparametric methods over their parametric counterpart.

The results for Task 2 show less discriminative differences, especially when using the ARMA approach. WT succeeds in identifying weak spectral differences within the alpha band (8–13 Hz) for a number of channels in the left frontal area. Notice that in all cases the discrimination levels achieved by either method are weak and do not support any significant differentiation between the studied populations in this task. The global FT method has also found the left brain area to show significant differences between the epileptic and the control group especially in the alpha and beta band, but once again the classification levels (Figure 5) are quite low.

**Figure 6:**
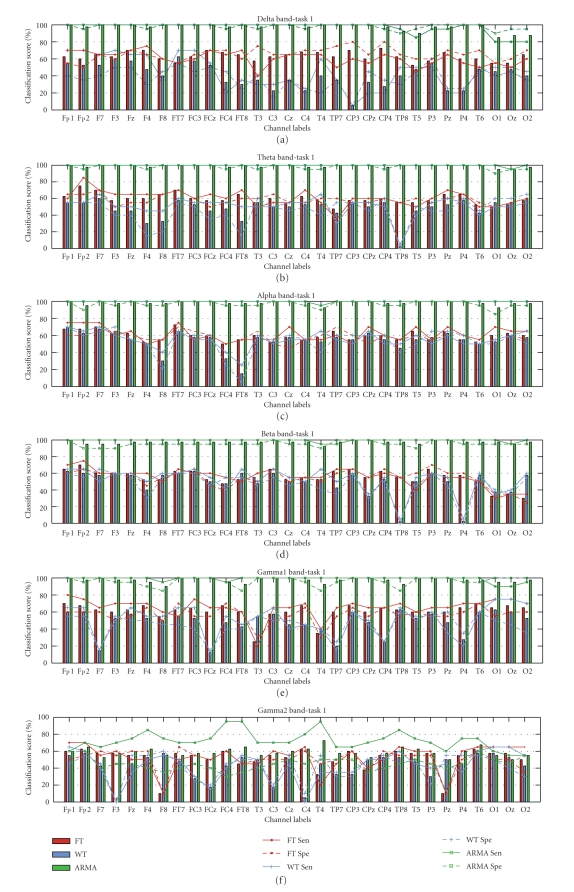
Classification scores, sensitivity, and specificity results for Task 1.

**Figure 7:**
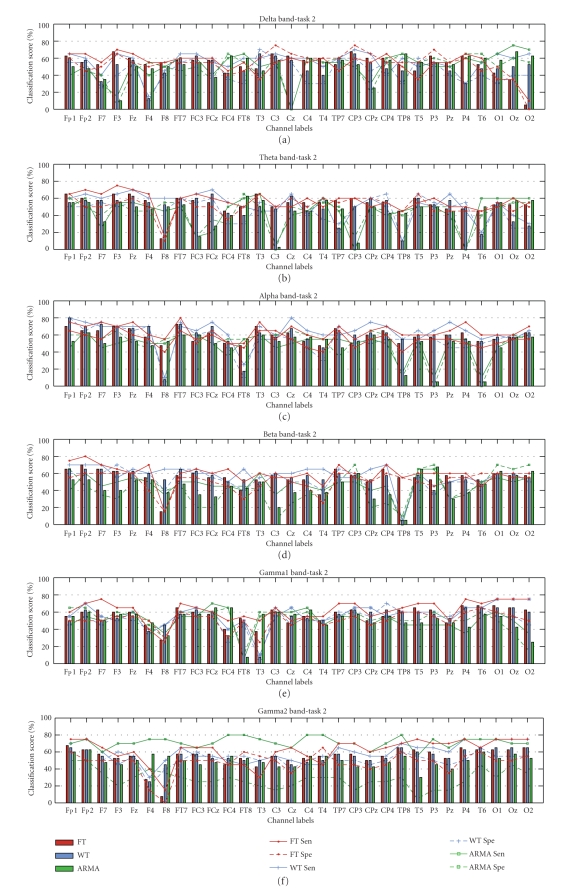
Classification scores, sensitivity and specificity results for Task 2.

The classification scores for both Tasks 1 and 2 are shown in Figure 5 as topographic maps; the complete results in the form of graph bars are presented in Figures 6 and 7. A linear discriminant analysis (LDA) classifier with the leave-one-out cross-validation scheme was implemented to derive the number of correctly classified subjects. The topographic maps depicted in Figure 5 present classification scores above 65% only. The topographic maps for classification scores present strong similarities with those for statistical significance, justifying that better discrimination of populations results in increased classification ability. The results for Task 1 obtained using the ARMA approach show that the highest scores occur over the left brain area for most bands and shift more towards the posterior for the beta and gamma1 band. For the WT approach, the topographic maps of classification scores show a concentration similar to that for the *p*-values, where only the alpha band shows a discriminating brain area between the two populations. FT gives more channels within the different bands with scores greater than 65% but similar to the WT, these scores are still significantly lower than those of ARMA. For Task 2, the classification scores for both the nonparametric and the parametric are sporadic. FT gave the largest number of locations within each band that can possibly discriminate between the two populations but the classification scores are still low and hence no conclusions can be derived concerning population differences for this more complex mathematical task.

Both Figures 6 and 7 show the scores for all channels together with the sensitivity and specificity measures for the two approaches. These results indicate more clearly that for the control task the classification scores for the ARMA parametric approach are significantly higher than those of the nonparametric approaches for all channels within the delta to the gamma1 band. With this technique, classification scores up to 100% were achieved on most channels (except gamma2 band). The sensitivity and specificity values for the ARMA technique are close to 100% implying that the ARMA approach can detect practically all epileptic and control subjects.

For Task 2, the classification scores are much lower and the differences between the parametric and nonparametric approaches are not as clear as for Task 1. The highest classification score of 80% was obtained by the WT approach over the frontal channels within the alpha band, particularly over Fp1, which was also found to be significant (*p* < 0.1) for this approach. The average classification score over all channels in the different frequency bands was in the range of 50%–60% but as shown earlier in the topographic maps and classification scores, the results for this task are quite random and hence nonconclusive.

**Figure 8:**
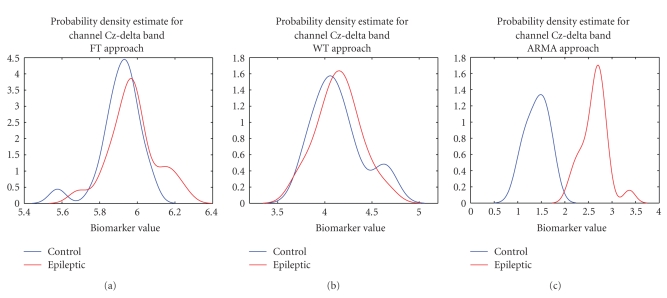
Power spectral feature distributions for control and epileptic subjects over channel Cz. Features were extracted from the delta band when the subjects were performing the control (rest) Task 1: (a) shows the results for the FT approach, (b) shows the results for the WT approach, and (c) shows the results for the ARMA approach.

In order to identify further differences in the feature distributions of the two subject groups, probability density estimates of the feature values of the patients and controls over different frequency bands and channel locations were also computed. Figure 8 shows the distributions of the feature values for Task 1 obtained over channel Cz when considering the delta band. The density plots for the control and epileptic subjects, for both the FT approach (Figure 8(a)) and the WT approach (Figure 8(b)), respectively, overlap significantly; hence the low-classification scores obtained using these nonparametric techniques. The results for ARMA, however, reached 100% classification over this channel and this is marked by the biomarker distribution plots (Figure 8(c)) which in this case are clearly separable. These density plots also show that the control group has a lower power spectral mean than the epileptic group. When the ARMA biomarkers obtained for the control task for the two subject groups were averaged across channels and compared, the results showed that epileptic patients have higher spectral power in all bands except the gamma2 band where the result was found to be insignificant.

**Figure 9:**
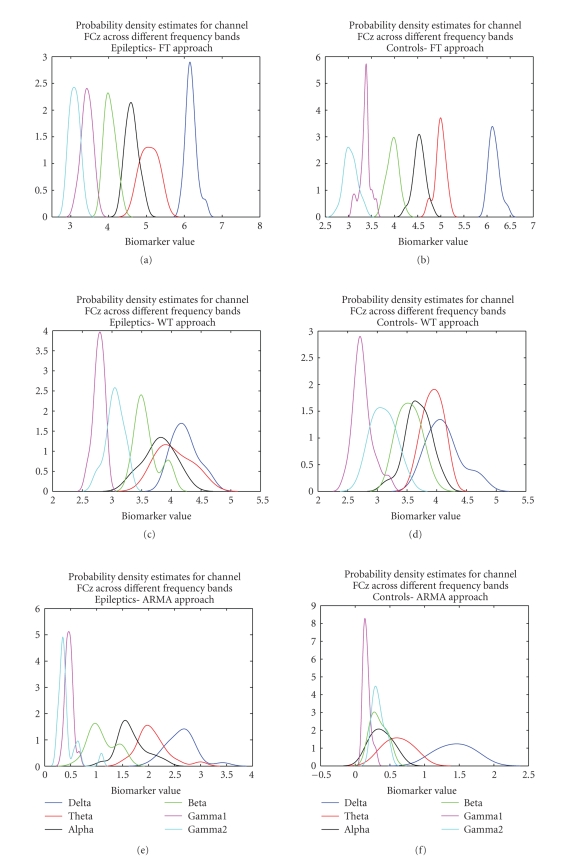
Power spectral feature distributions for epileptics and controls comparing the differences between the different frequency bands. (a) and (b) show the results for FT, (c) and (d) show the results for wavelets, and (e) and (f) show the results for ARMA.

The set of plots shown in Figure 9 describe the variations in the probability density functions of epileptics and controls, respectively, across the six different frequency bands considered. For brevity, only the results across channel FCz for Task 1 are shown. The plots for the parametric technique (Figures 9(e) and 9(f)) show that the biomarker for the epileptic subjects is generally of higher value than that of the control subjects. Furthermore the spread of the density estimates is also slightly higher for epileptics than controls. For the nonparametric techniques (Figures 9(a)–9(d)) this trend is not clearly visible and within practically all bands there is a substantial overlap between the plots of the two populations. This distribution of biomarker values explains the classification scores discussed earlier.

## 4. Discussion

This work considers methods for the discrimination of two groups of age-matched children, that is, controls and children with controlled epilepsy. Initial clinical and psychological examinations, as well as visual EEG inspection, do not provide any information leading to possible differences. On the original EEG data we apply two types of methodologies, one based on direct signal representation (through nonparametric techniques, mainly the Fourier transform and the wavelet transform) and the other at modeling the signal dynamics (through a parametric ARMA model). The spectral features extracted by these methods in each channel and spectral band are examined through significance tests, classification accuracies, and statistical distributions of biomarkers. This work indicates that parametric modeling of the EEG dynamics provides better representation of the significant EEG content than nonparametric techniques for feature extraction. The features extracted by the ARMA model for the control task provide higher discrimination power than those extracted by the Fourier transform and wavelet approaches. The results for the Fourier transform have shown to be slightly superior than those of the wavelet transform in this case where the biomarker is an average of the spectral power over the whole period of data. This may be the cause of artefacts introduced by the windowing leakage effect of wavelets which is less dominant in the global Fourier transform approach where a single window was considered. In other situations where the temporal resolution is taken into consideration, it is expected that wavelets outperform the Fourier transform technique.

Comparing the control and math tasks, the methods derive significant differences during the control (rest) task, but they are unable to identify any consistent differences during the more demanding mathematical task where the discrimination of the specific brain dysfunction seems more difficult.

The potential clinical benefit of this work is the analysis of EEG data towards the identification of children with mild epilepsy at early stages, where classical, neurological, and clinical examinations and detailed psychological and neuropsychological testing are unable to identify any signs of brain dysfunction. The ARMA results show that epileptic children during the control task have higher activity in frequency bands up to the gamma1 band, but this activity becomes similar in both groups for frequencies within the gamma2 band. When analyzing an adult-patient group, Tuunainen et al. [7] have also found higher spectral activity in epileptic patients. In particular, their results showed that the absolute power of the epileptic group over all electrode sites was significantly higher at baseline than in controls for delta, theta, beta, and total activity. Absolute alpha power was also higher but this was not found to be significant. In a similar study on adult patients, Willoughby et al. [2] illustrated that patients with partial generalized epilepsy have higher power at 3–7 Hz centrally, 15–17 Hz anteriorly and over 25 Hz in all channels. Finally, in a preliminary study on children, increased spectral power was also found in the theta and alpha bands [35]. The increased spectral power can be attributed either to the age-group being analyzed , or the type of epilepsy and the level of brain dysfunction, or to drug effects. AEDs are known to result in higher power within the lower-frequency bands [2]. But since in this analysis the drugs provided were in low dosage, it is most probable that the differences are signs of brain dysfunction for such a typical child population, which in turn fade out when intense mental thinking (during the mathematical task) takes over. Furthermore, if AEDs do influence the EEG signal structure (because of high dosages), they will be identified as a consistent pattern apparent in every electrode. No such effect is known to occur with the AEDs used in our case and, if any, it would be expected to be diffused and affecting only slow waves [36].

## 5. Conclusion

This work involved the study of children with mild epilepsy who had epileptic seizures in the past but who did not exhibit any clinical, physiological, or visible neurophysiological symptoms during the study. The goal of this analysis was to develop reliable techniques to test if such controlled epileptic conditions induce related spectral differences in the EEG. The results show that parametric ARMA modeling techniques extract more reliable biomarkers than the nonparametric Fourier and wavelet transform techniques implemented here. For the control task, the ARMA technique led to classification scores up to 100% across all channels for frequency bands ranging from the delta to the gamma1 band.

Diagnosis of epilepsy was here conducted by considering biomarkers on an average of the spectral power over the whole 10.24-second period of data available. Future work will investigate whether taking into account the temporal information enhances these classification scores specifically for the math task where the complexity of the task made it difficult to capture any brain dysfunction through global biomarkers. In the latter case it is expected that wavelets will outperform the Fourier transform technique and lead to results which are comparable to its parametric counterpart.

## References

[1] Olesen J, Baker MG, Freund T (2006). Consensus document of European brain research. *Journal of Neurology, Neurosurgery, and Psychiatry*.

[2] Willoughby JO, Fitzgibbon SP, Pope KJ (2003). Persistent abnormality detected in the non-ictal electroencephalogram in primary generalised epilepsy. *Journal of Neurology, Neurosurgery, and Psychiatry*.

[3] Larsson PG, Kostov H (2005). Lower frequency variability in the alpha activity in EEG among patients with epilepsy. *Clinical Neurophysiology*.

[4] Hongou K, Konishi T, Naganuma Y, Murakami M, Yamatani M, Okada T (1993). Development of the background activity of EEG in children with epilepsy; 
comparison with normal children. *No To Hattatsu*.

[5] Klimesch W (1999). EEG alpha and theta oscillations reflect cognitive and memory performance: a review and analysis. *Brain Research Reviews*.

[6] Salinsky MC, Oken BS, Storzbach D, Dodrill CB (2003). Assessment of CNS effects of antiepileptic drugs by using quantitative EEG measures. *Epilepsia*.

[7] Tuunainen A, Nousiainen U, Pilke A, Mervaala E, Partanen J, Riekkinen P (1995). Spectral EEG during short-term discontinuation of antiepileptic medication in partial epilepsy. *Epilepsia*.

[8] Ferri R, Curzi-Dascalova L, Arzimanoglou A (2002). Heart rate variability during sleep in children with partial epilepsy. *Journal of Sleep Research*.

[9] Gath I, Feuerstein C, Pham DT, Rondouin G (1992). On the tracking of rapid dynamic changes in seizure EEG. *IEEE Transactions on Biomedical Engineering*.

[10] Jerger KK, Netoff TI, Francis JT (2001). Early seizure detection. *Journal of Clinical Neurophysiology*.

[11] Blanco S, Kochen S, Rosso OA, Salgado P (1997). Applying time-frequency analysis to seizure EEG activity. *IEEE Engineering in Medicine and Biology Magazine*.

[12] Williams WJ, Zaveri HP, Sackellares JC (1995). Time-frequency analysis of electrophysiology signals in epilepsy. *IEEE Engineering in Medicine and Biology Magazine*.

[13] Kalayci T, Ozdamar O (1995). Wavelet preprocessing for automated neural network detection of EEG spikes. *IEEE Engineering in Medicine and Biology Magazine*.

[14] Yaylali I, Kocak H, Jayakar P (1996). Detection of seizures from small samples using nonlinear dynamic system theory. *IEEE Transactions on Biomedical Engineering*.

[15] Shamsollahi MB, Senhadji L, Le Bouquin-Jeannes R Detection and localization of complex SEEG patterns in epileptic seizures using time-frequency analysis.

[16] Zapata-Ferrer A, Maya LR, Gonzalez AG (1999). Detecting the onset of epileptic seizures. *IEEE Engineering in Medicine and Biology Magazine*.

[17] Niedermeyer E, Niedermeyer E, Lopes da Silva F (1998). Epileptic seizure disorders. *Electroencephalography: Basic Principles, Clinical Applications, and Related Fields*.

[18] Wolf P (1994). *Epileptic Seizures and Syndromes*.

[19] Lagae L (2006). Cognitive side effects of anti-epileptic drugs. The relevance in childhood epilepsy. *Seizure*.

[20] von Aster M, Weinhold M (2002). *Neuropsychologische Testbatterie für Zahlenverarbeitung und Rechnen bei Kindern (ZAREKI)*.

[21] Kiymik MK, Güler I, Dizibüyük A, Akin M (2005). Comparison of STFT and wavelet transform methods in determining epileptic seizure activity in EEG signals for real-time application. *Computers in Biology and Medicine*.

[22] Tarvainen MP, Hiltunen JK, Ranta-Aho PO, Karjalainen PA (2004). Estimation of nonstationary EEG with Kalman smoother approach: an application to event-related synchronization (ERS). *IEEE Transactions on Biomedical Engineering*.

[23] Aboy M, McNames J, Marquez OW, Hornero R, Thong T, Goldstein B Power spectral density estimation and tracking of nonstationary pressure signals based on Kalman filtering.

[24] Bracewell RN (2000). *The Fourier Transform and Its Applications*.

[25] Burrus CS, Copinath RA, Gao H (1998). *Introduction to Wavelets and Wavelet Transforms: A Primer*.

[26] Sakkalis V, Zervakis M, Micheloyannis S (2006). Significant EEG features involved in mathematical reasoning: evidence from wavelet analysis. *Brain Topography*.

[27] Rosso OA, Martin MT, Figliola A, Keller K, Plastino A (2006). EEG analysis using wavelet-based information tools. *Journal of Neuroscience Methods*.

[28] Torrence C, Compo GP (1998). A practical guide to wavelet analysis. *Bulletin of the American Meteorological Society*.

[29] http://www.documents.wolfram.com/applications/timeseries/UsersGuidetoTimeSeries/1.2.1.html.

[30] Priestley MB (1981). *Spectral Analysis and Time Series Vol. 1*.

[31] Zar JH (1999). *Biostatistical Analysis*.

[32] Field AP (2005). *Discovering Statistics Using SPSS*.

[33] Krzanowski WJ (1988). *Principles of Multivariate Analysis*.

[34] Sakkalis V, Zervakis M, Bigan C Validation of time-frequency and ARMA feature extraction methods in classification of 
mild epileptic signal patterns.

[35] Martin-Fiori E Thalamo-cortical Oscillation during Brain Development and in Epilepsy. http://www.forschungsportal.ch/unizh/media/pdf/p8421.pdf.

[36] Macdonald RL (1989). Antiepileptic drug actions. *Epilepsia*.

